# Adverse events of pancreatic extracorporeal shock wave lithotripsy: a literature review

**DOI:** 10.1186/s12876-023-02992-0

**Published:** 2023-10-18

**Authors:** Jin-Hui Yi, Zhao-Shen Li, Liang-Hao Hu

**Affiliations:** Department of Gastroenterology, Shanghai Changhai Hospital, 168 Changhai Road, Shanghai, 200433 China

**Keywords:** Pancreatic stones, Chronic Pancreatitis, Pancreatic extracorporeal shock wave lithotripsy, Adverse events, Endoscopy

## Abstract

Pancreatic stones are the result of pathophysiologic changes in chronic pancreatitis with an incidence of more than 90%. At present, pancreatic extracorporeal shock wave lithotripsy (P-ESWL) can be used as the first-line treatment for large or complex stones. Although a large number of studies have proven the safety and effectiveness of P-ESWL, we should also pay attention to postoperative adverse events, mainly due to the scattering of shock waves in the conduction pathway. Adverse events can be classified as either complications or transient adverse events according to the severity. Because the anatomic location of organs along the shock wave conducting pathway differs greatly, adverse events after P-ESWL are varied and difficult to predict. This paper outlines the mechanism, definition, classification, management and risk factors for adverse events related to P-ESWL. It also discusses the technique of P-ESWL, indications and contraindications of P-ESWL, and adverse events in special populations.

## Introduction

Chronic pancreatitis (CP), usually caused by alcohol abuse, smoking, or certain gene mutations, is characterized by irreversible destruction of pancreatic parenchyma, inflammatory cell infiltration and progressive fibrosis of pancreatic tissue, which is eventually followed by recurrent attacks of painful pancreatitis or other manifestations of endocrine or exocrine pancreas secretion dysfunction. Pancreatic stone formation is a common pathological change in the course of CP with an incidence of over 90% [1]. These stones tend to cause further pancreatic duct obstruction, pancreatic parenchymal hypertension and ischaemia. Therefore, removing pancreatic stones is the core to effectively relieve CP symptoms. Endoscopic retrograde cholangiopancreatography (ERCP) is the first choice of the minimally invasive methods. However, ERCP may not succeed if stones are large or complex, while pancreatic extracorporeal shock wave lithotripsy (P-ESWL), which has been applied since 1987, could overcome this problem [2].

At present, a large number of studies have confirmed the safety and efficacy of P-ESWL, but the rates of adverse events in these studies are highly variable, ranging from 0 to 63% [3–6]. These varying results have resulted from the use of a variety of lithotripters, different shock energy and number of shock waves, methods of anaesthesia, and, finally, from a lack of uniform criteria for measuring the adverse events.

This paper reviews and summarizes the current literature on the adverse effects of P-ESWL. It outlines the mechanism, definition, classification, management and risk factors for adverse events related to P-ESWL. It also discusses the technique of P-ESWL, indications and contraindications of P-ESWL, and the adverse events in special populations.

## Indications and contraindications of P-ESWL

According to guidelines by various societies, P-ESWL is recommended for the clearance of radiopaque obstructive main pancreatic duct (MPD) stones larger than 5 mm located in the head/body of the pancreas [7–11].

The contraindications of P-ESWL include noncorrectable coagulation disorders, pregnancy, and presence in the shockwave path of bone, calcified vessels, or lung tissue. Patients with implantable defibrillators and pacemakers should receive specific precautions [7].

## P-ESWL procedure

Lithotripters contain four components: a shock wave generator; a means of coupling the shock wave to the patient; a focusing system and an imaging modality to target the stone, such as fluoroscopy or ultrasound [12]. Shock waves that are generated outside the body by a lithotripter fragment the stones within the body [13]. Lithotripsy machines can be divided into electrohydraulic, electromagnetic or piezoelectric shock-wave-generating devices [14]. An electrohydraulic lithotripter is rarely used at present due to its large damage to tissues and frequent equipment repair. Electromagnetic or piezoelectric shock-wave-generating devices are commonly used now, but piezoelectric lithotripters are not as widely used as electromagnetic lithotripters because they have lower energy levels and stone fragmentation rates.

Compared to the early application of P-ESWL, where patients were immersed in a water bath and shock waves entered the body from the rear, patients are now placed in the supine position with the shock head touching the abdominal skin of the right upper quadrant from above, and the shock wave path is at a 45° angle to the ventral midline. Sometimes, patients are tilted to one side by placing a bolster below the back to achieve effective contact with the shock wave head [14]. Patients are treated under epidural anaesthesia or general anaesthesia in most centres due to the shock waves at large energy levels causing too much pain, but target-controlled infusion of remifentanil with flurbiprofen axetil has also been verified as a satisfactory analgesia for P-ESWL [6, 15, 16].

To determine the developments of technological models of P-ESWL, we found 26 articles with the simultaneous description of lithotripsy machines, intensity energy and the number of shock waves per session in PubMed since the first use of P-ESWL in 1987 (Table 1).

**Table 1:** Study characteristics of pancreatic extracorporeal shock wave lithotripsy

Author, Year	Country	Sample size^1^	Lithotripters^2^	Intensity	Frequency (shock waves/min)	Number of shock waves per session	Treatment time per session (min)	anaesthesia means^3^
Sauerbruch et al. 1987 [2]	Germany	1	Dornier HM3	18 KV	N/A	1200	40	GA
Sauerbruch et al. 1989 [52]	Germany	8	Dornier HM3	18 kV	N/A	1356	36	GA or IA
Kerzel et al. 1989 [53]	Germany	1	Wolf Piezolith 2300	Levels III-IV ^4^	N/A	5600	45	WOA
Delhaye et al. 1992 [3]	Belgium	123	Siemens Lithostar	10-19KV	100	2862	60	IA
Sauerbruch et al. 1992 [54]	Germany	24	Dornier HM3	18-24 kV	N/A	1780	30–60	GA or IA
Van Der Hul et al. 1994 [55]	Netherland	17	Siemens Lithostar	16.2-19KV	N/A	3000–6000	N/A	IA
Martin et al. 1995 [56]	USA	6	Dornier HM4	18–24 KV	N/A	1200–2400	N/A	IA
Wolf et al. 1995 [57]	USA	14	Dornier HM3	20 KV	N/A	2000	N/A	IA
Schreiber et al. 1996 [58]	Austria	10	Dornier MPL 9000	19 KV	N/A	750	44	IA
Johanns et al. 1996 [4]	Germany	35	Dornier MPL 9000	14-22KV	N/A	2000	N/A	IA
Adamek et al. 1999 [59]	Germany	80	Wolf Piezolith 2300	Levels III-IV ^4^	N/A	3500	60	IA
Karasawa et al. 2002 [60]	Japan	24	Wolf Piezolith 2500	Levels III-IV^4^	N/A	4200	N/A	WOA
Kozarek et al. 2002 [5]	USA	40	Dornier HM3	18–24 KV	N/A	1800–2400	N/A	GA or EA
Lawrence et al.2010 [61]	USA	29	Storz Modulith SLX	7–8 KV	N/A	3000–6000	N/A	GA
Tandan et al. 2010 [6]	India	1006	Dornier Delta Compact	15-16KV	90	5000	60–90	EA
Milovic et al. 2011 [62]	Germany	32	Storz Minilith SL 1	adjusted to the individual^5^	N/A	6800	N/A	WOA
Merrill et al. 2011 [63]	USA	30	Dornier HM3 Storz Modulith SLX-F2	Levels 3–9^4^ Levels 3–9^4^	90–120 90–120	3000–5000 3000–5000	N/A N/A	GA GA
Li et al. 2014 [17]	China	634	Dornier Compact Delta II	10-16KV	60–120	5000	60–90	IA
Hu et al. 2016 [15]	China	214	Dornier Compact Delta II	16KV	100	5000	60–90	IA
Vaysse et al. 2016 [64]	France	146	Dornier Delta Compact	adjusted to the individual^5^	100	1200–6000	N/A	IA
Tandan et al. 2019 [18]	India	5124	Dornier Delta Compact	15-16KV	90	5000–6000	N/A	EA
Korpela et al. 2016 [65]	Finland	83	Storz Modulith SLX Storz Modulith SLX-F2	Levels 6^4^ Levels 4^4^	60–90 60–90	3000 3000	N/A N/A	N/A N/A
Lapp et al. 2016 [66]	USA	37	Wolf Piezolith 3000	18KV	N/A	2500	N/A	N/A
Hao et al. 2019 [47]	China	1404	Dornier Compact Delta II	16KV	120	5000	60–90	IA
Liu et al. 2019 [67]	China	106	Dornier Compact Delta II	16KV	100	5000	60–90	IA
Hyun et al. 2021 [68]	USA	97	Storz Modulith SLX-F2	Levels 6–7^4^	N/A	3000–5500	N/A	GA

^1^Some studies have sample size overlaps: The sample size of Reference 47 is included in Reference 48; The sample size of Reference 6 is included in Reference 14; The sample size of Reference 11 is included in Reference 13, and they are all included in Reference 41

^2^The type of lithotripter is represented by company name and machine model

^3^ GA: general anaesthesia; IA: intravenous anaesthesia; EA: epidural anaesthesia; WOA: without anaesthesia

^4^Only energy level settings are available in studies, and specific energy parameters are unknown

^5^ “adjusted to the individual” means energy level or number of shock waves are tailored to the individual pain tolerance of the patient

Since P-ESWL began to be applied, higher intensity energy than urinary ESWL has become the dominant model in the world, which is reasonable because pancreatic stones are hard and difficult to pulverize by low intensity energy. Low intensity or adjusting intensity tailored to the individual pain has also been reported occasionally. With the improvement of lithotripsy machines and the development of medical technology, the number of shock waves per session has gradually increased. There are a large number of lithotripsy machines provided by different companies used in P-ESWL. We think that no matter what lithotripter is adopted, it is effective as long as the intensity energy can fragment pancreatic stones. Since 2000, nearly 95% of the P-ESWL procedures reported in the studies have been performed by a third-generation electromagnetic lithotripter (Delta Compact or Compact Delta II) provided by Dornier Med Tech. Shock waves up to a maximum of 5000–6000 shocks are delivered per sitting, and an intensity of 15–16 KV is used with a frequency of 90–120 shocks per minute during the procedure. The duration of each session was 60 to 90 min. The second most common lithotripsy machine is also an electromagnetic lithotripter (Modulith SLX, SL 1 or SLX-F2) provided by Storz Medical AG.

## Mechanism of adverse events

The mechanisms of adverse events may be as follows. First, the energy of the shock wave will be released before reaching the target stones, which will damage the organs along the shock wave conduction pathway. Second, although we try to localize the stones in the focal point, the position of stones always changes with the respiratory motion. This inaccurate targeting results in part of the energy being released around the stones rather than hitting the stones precisely. Third, when intravenous analgesia is used for analgesia and sedation, the involuntary movement of patients would lead to stone location bias and adjacent tissue damage. Because the anatomic location of organs along the shock wave conducting pathway differs greatly, adverse events after P-ESWL are varied and difficult to predict.

## Definition and classification of adverse events

In 2014, Li and coworkers first proposed a criterion for post-ESWL adverse events. This criterion provides guidelines for the management based on hospitalization days and the interventions required to treat adverse events [17]. According to the severity, adverse events can be classified as either complications or transient adverse events (TAEs).

TAEs refer to transient and reversible injuries caused by shock waves, which require no medical intervention and do not prolong hospitalization, and they include symptoms, such as skin erythema, mild tenderness of the region in contact with the shockwave head, asymptomatic hyperamylasemia, haematuria, and acute gastrointestinal mucosal injury (manifested as haematemesis and melena). Asymptomatic hyperamylasemia is defined as an increase in serum amylase compared with the pre-ESWL levels and beyond the upper limit of the normal range but showing no related symptoms [17]. According to the studies from America, the rate of TAEs after P-ESWL is approximately 15%, and most cases are skin erythema [5]. In India, skin erythema and pain at the site of delivery of shocks are common reports, with incidences of 19% and 13.5%, respectively [18]. In China, the rate of TAEs is approximately 21.2%, and asymptomatic hyperamylasemia is the most common TAE, with a rate of 15.5%. The rate of haematuria is approximately 4.2%. The prevalence of acute gastrointestinal mucosal injury is 2.7% after P-ESWL [17].

Complications are characterized as adverse events needing specific medical intervention and prolonged hospitalization and are classified into five groups: post-ESWL pancreatitis, bleeding, infection, steinstrasse and perforation. In addition, some rare complications have been reported but not included in this classification of complications. According to the length of hospitalization days and subsequent treatment, each group of complications can also be classified as mild, moderate or severe (Table 2) [17]. A nationwide survey in Japan showed that acute pancreatitis is the most common complication, with a risk of 4.4% [19]. In America, post-ESWL pancreatitis and bleeding are common complications with the same rate of 2.5% [5]. In India, post-ESWL pancreatitis can be seen in 3.6% of patients, and 0.5% of patients require hospitalization for more than 3 days [18]. In China, the overall complication rate is approximately 6.73%, with incidences of post-ESWL pancreatitis, infection, steinstrasse, bleeding and perforation of 4.35%, 1.4%, 0.4%, 0.3% and 0.3%, respectively [17].

**Table 2:** Definitions of major complications of pancreatic extracorporeal shock wave lithotripsy [17]

Complication^1^	Mild	Moderate	Severe
Post-ESWL pancreatitis	Clinical pancreatitis, amylase at least three times the normal level at >24 h after procedures, require admission or extension of planned admission from 2 days to 3 days	Requires hospitalization of 4–10 days	Hospitalization for 10 days, pseudocyst or intervention (percutaneous drainage or surgery)
Bleeding^2^	Clinical evidence of bleeding, hemoglobin drop<3 g, no transfusion	Transfusion of ≤ 4 units, no angiographic intervention, or surgery	Transfusion of ≥ 5 units or intervention (angiographic or surgery)
Infection	>38℃ for 24–48 h	Require >3 days of hospital treatment	Abscess, septic shock, or intervention (percutaneous drainage or surgery)
Steinstrasse	Severe abdomen pain without other post-ESWL complications	Combined with other complications, or requires >3 days of hospital treatment	Combined with other complications; hospitalization>10 days, or surgery
Perforation	Possible, or very slight leak of fluid, treatable with fluids and suction for ≤ 3 days	Any definite perforation treated medically for 4–10 days	Medical treatment for >10 days or intervention (percutaneous or surgical)

ESWL: extracorporeal shock wave lithotripsy

^1^ Splenic rupture, pancreaticobiliary fistula, and other rare complications are not included in this classification of complications

^2^ Acute gastrointestinal mucosal injury was not included; it was classified as a transient adverse event

## Manifestations and management of Complications

Post-ESWL pancreatitis is the most common complication after P-ESWL, which may be caused by the direct damage of shock waves or hypertension of the pancreatic duct due to stone fragments. Different from noniatrogenic acute pancreatitis, which is graded by clinical manifestations and prognosis, post-ESWL pancreatitis is classified into mild, moderate or severe based on the Cotton criteria, but the clinical manifestations, diagnosis and treatment strategy are similar to those of noniatrogenic acute pancreatitis [20]. In addition, post-ESWL pancreatitis cannot be distinguished from perforation, splenic rupture or superficial tissue injury based on abdominal pain alone, and computed tomography (CT) scans should be performed for differentiation.

How to prevent post-ESWL pancreatitis has become a research focus in recent years. Due to the conclusion that pancreatic stenting prior to ERCP can effectively prevent post-ERCP pancreatitis, Japanese researchers have tried to implant pancreatic stents before P-ESWL. The stenting group tended to have a lower frequency of pancreatitis than the nonstenting group (2.2% vs. 11.3%, p = 0.162) [21]. However, other researchers consider that pancreatic stenting will not only increase medical costs but also affect the process of spontaneous clearance of pancreatic stones after adequate fragmentation by P-ESWL. In 2022, Qian designed a double-blind, randomized, placebo-controlled trial. A total of 1370 patients with pancreatic stones (> 5 mm in diameter) were enrolled, 685 patients were randomly assigned to receive 100 mg rectal indomethacin 30 min before P-ESWL, while the other 685 patients were randomly assigned to receive identical glycerin (placebo) suppositories. Post-ESWL pancreatitis occurred in 9% of patients in the rectal indomethacin group and 12% of patients in the placebo group (P = 0.042). This study indicated that preprocedural administration of rectal indomethacin is an efficacious and safe means of reducing the incidence of post-ESWL pancreatitis [22].

Infection usually occurred within a few hours after P-ESWL. The major pathogenesis is bacteraemia caused by intestinal mucosal barrier damage, after which bacteria enter the blood. The clinical manifestations are hyperthermia, chills, remittent fever or continued fever, and the blood culture result usually being *Escherichia coli*. For these patients, broad-spectrum antibiotic therapy at an early stage is recommended, and effective antibiotics should be selected based on the blood culture result or antibacterial susceptibility test. In addition, there are still a small number of patients with delayed splenic abscess, severe cases can develop into sepsis or peritonitis, for which splenectomy has to be performed, abscess drainage or puncture catheter can also become a treatment choice according to the change of illness condition [23]. Common bile duct stricture can occur as a consequence of pancreatic parenchymal oedema after P-ESWL, which may be associated with a high risk of developing cholangitis or sepsis. Patients who have transient pain or jaundice can be treated conservatively. Duodenal sphincterotomy or endoscopic stenting is advised when there is persistent jaundice or hyperthermia, and surgery is necessary when endoscopic treatments fail [24, 25].

Steinstrasse was previously defined as a post-ESWL complication of the urinary tract stones, with partial or complete ureteral block caused by stone fragments to form a “stone street”, which often superimposed with infection or renal failure [26]. Hu et al. first described this rare complication after P-ESWL in 2012 [27]. In reference to the definition of steinstrasse in urinary ESWL, researchers defined steinstrasse after P-ESWL as acute stone incarceration in the papilla that leads to poor pancreatic juice drainage and CT findings of dilated pancreatic duct with/without acute pancreatitis [17]. Obviously, there are three simultaneous reasons for steinstrasse occurrence: severe stricture of the pancreatic duct, a large number of stone fragments and a larger performance area of P-ESWL than expected due to position bias. The main manifestation is severe abdominal pain that cannot be eased by analgesics, which should be relieved by emergency ERCP. Pancreatic sphincter precutting using a dual knife can be performed if the catheter is impassable due to a swollen papilla [28].

Different from post-ERCP gastrointestinal bleeding, such as duodenal bulb injury or postsphincterotomy bleeding, post-ESWL bleeding is defined as bleeding in a closed chamber due to shock wave damage in peripancreatic organs, including hepatic subcapsular haematoma, mesenteric haematoma, colonic haematoma, gastric submucosal haematoma and renal subcapsular haemorrhage [29–33]. Bleeding often occurs immediately or within a few hours after P-ESWL. During the P-ESWL procedure, shock waves are targeted to pancreatic stones so that less force spreads to adjacent tissue and the bleeding is limited to be within a closed cavity. Conservative treatment is advised to be the first-line treatment based on closely monitoring the vital signs, whereas digital subtraction angiography (DSA) or an emergency operation should be performed when conservative treatment fails.

The underlying reason for perforation is the large acoustic impedance difference between normal tissue and gas or faeces in the gastrointestinal tract, for which the shock wave will release more energy. Although the stomach wall and feasible intestine are difficult to injure, a relatively fixed hepatic flexure is more likely to be damaged, which is different from post-ERCP perforation. Perforation in the duodenum is possible but has not yet been reported. Leakage is usually due to intestinal juice and gas, which contribute to obvious peritonitis symptoms, but the pores are too small to be detected by colonoscopy. Physical examination showed board-like rigidity, and standing abdominal plain film radiography indicated the presence of subdiaphragmatic free air. For these patients, continuous gastrointestinal decompression is the core of treatment, supplemented by somatostatin or octreotide to reduce the secretion of intestinal juice and to keep the gastrointestinal tract clean. Conservative treatments can relieve the symptoms in most patients, whereas laparoscopic treatments are strongly recommended if the patient’s condition deteriorates (presenting with septic or peritonitis signs) [34, 35].

## Other rare complications

The peripancreatic organs, such as the spleen [36], lung and kidney [37], are positioned, at least partly, in the way of the energy path, and solid structures such as the vertebrae and ribs or even a firm, fibrotic pancreas may redirect part of the shock wave energy towards these tissues, which may result in injury to them. Patients can complain of corresponding symptoms and signs, including abdominal pain, cough, haemoptysis, hypoxemia or haematuria. However, a large number of affected patients go undetected because they heal mildly or their conditions are confused with other diseases.

Haemorrhagic pseudoaneurysm in a pancreatic pseudocyst after P-ESWL was reported in 2011 [38]. Enzyme-rich peripancreatic fluid in the pseudocyst causes autodigestion and weakening of the walls of the adjacent vessels (splenic veins and artery) and then stimulates peritoneal fibrous hyperplasia and encapsulation, resulting in pseudoaneurysm formation [39]. In addition, cellular injury and ultrastructural damage, caused by P-ESWL, also induced the developed of pseudoaneurysms. A comprehensive review of the literature reported that haemorrhagic pseudoaneurysm in a pancreatic pseudocyst is the most rapidly fatal complication of pancreatitis, with a mortality of 18-29% in operative patients and over and a mortality over 90% when patients receive nonoperative supportive measures alone [40]. Li et al. verified that P-ESWL is a safe means in patients with coexisting pancreatic stones and pancreatic pseudocysts, but pancreatic portal hypertension and noncommunicating pancreatic pseudocysts may be attributed to the high risk of P-ESWL complications [41].

A fistula can be formed by the force of stone collapse. If the pancreatic duct is injured alone, a pancreatic fistula occurs and is generally detected when the guide wire passes out of the pancreatic duct during ERCP after P-ESWL. A small fistula can close spontaneously, and most patients complain of no obvious symptoms without special treatments. However, when both the pancreatic duct and bile duct are injured, a pancreaticobiliary fistula forms, and typical pancreatic stones are found in the bile duct. Moreover, the other possible mechanism of pancreaticobiliary fistula formation may be that stasis of pancreatic juice induced by a branched intraductal stone would in turn directly injure the bile duct wall, resulting in pancreaticobiliary fistula formation. These pancreaticobiliary fistulas are often detected after fragmenting stones [42].

Shock waves can accelerate intestinal peristalsis, which induces the occurrence of intussusception. It has been reported that patients with obvious intestinal flatulence are prone to develop a transverse colon intussusception after P-ESWL, and it may lead to closed-loop obstruction because the ileocecal valve is unidirectional [43]. Therefore, early differential diagnosis between intussusception and other common complications is extremely important if the patient complains of abdominal pain or intestinal flatulence after P-ESWL.

## Risk factors for adverse events

For TAEs, the multivariate analysis showed five protective factors, including diabetes, steatorrhea, previous ERCP, needing further P-ESWL and multiple-location of targeted stones, while acute pancreatitis attack in 3 months and pseudocyst in chronic pancreatitis course were detected as risk factors [17].

Diabetes and steatorrhea, which are caused by tissue fibrosis and atrophy progression of pancreas, are protective factors for P-ESWL complications. The relatively lower complication rate may be explained by decreased enzymatic activity due to the increasing degree of fibrosis tissue [44]. The risk factors are female sex, pancreas divisum and a longer interval between the diagnosis of chronic pancreatitis and P-ESWL. Pancreatic duct stricture and previous treatments may not be associated with P-ESWL complications. Both dysfunction of the sphincter of Oddi and susceptibility to an inflammatory response to pancreatic damage are complications in females. In moderate-to-severe complications, female sex is also a risk factor. Pancreatic divisum is detected as a risk factor because the relatively narrower caliber of the accessory pancreatic duct and the minor papilla may expose patients to pancreatic juice outflow obstruction after P-ESWL. In post-ESWL pancreatitis, Li et al. considered that female sex, nonsteatorrhea, pancreas divisum and frequent attacks of acute pancreatitis are risk factors. A high frequency of acute episodes suggests that the patient has genetic disposition to acute pancreatitis and has a high enzymatic activity of the pancreas [17]. Ru et al. suggested that steatorrhea, multiple stones, and stones located at the head combined with the body or tail of the pancreas are independent protective factors for post-ESWL pancreatitis. The underlying of multiple and widely distributed stones becoming protective factors might be that they aggravate pathological changes in the pancreatic ducts and then worsen insufficient endocrine or exocrine pancreas functions [45].

For patients needing more sessions of P-ESWL, the decrease in the pancreatic stone volume and the partial obstruction release of the pancreatic duct may explain the lower adverse event rates in the second session than in the first session. Patients who undergo post-ESWL pancreatitis or asymptomatic hyperamylasemia in the first session are more likely to develop adverse events during subsequent sessions [17].

## Adverse events in special populations

A prospective observational study showed that no significant differences were observed in the complication type or rate when the same intensity of shock wave was applied in both adult and paediatric patients (11.1% vs. 12.8%, P = 0.68) [46].

Because most geriatric patients have endocrine and exocrine insufficiency, the incidence of complications may be lower than that in adult patients. Hao et al. found no significant differences between the geriatric and adult groups regarding the incidence of post-ESWL complications (8.3% vs. 11.9%, P = 0.364) [47].

For patients with a history of pancreatic surgery, the heterogeneity of acoustic impedance is increased by surgical scars, adhesions, and foreign bodies (such as staples). Moreover, reconstruction of the gastrointestinal tract changes the organs in the transduction pathway. However, significant differences were not observed between the surgical and matched controls (14.0% vs. 13.2%, P = 0.877), which can be explained by the significant difference in acoustic impedance between the stones and soft tissues (scars and adhesions) and accurate targets for stones [48].

## Discussion and conclusion

There have been a large number of in-depth studies on the mechanism, definition, classification, risk factors and management of post-ESWL adverse events in recent decades. Considering that P-ESWL is an effective and safe means and that most adverse events can be well controlled, many guidelines suggest that P-ESWL should be the first-line therapy as a nonsurgical intervention for main pancreatic duct stones in patients with chronic pancreatitis who do not receive adequate pain relief with conservative management [10].

However, there are some limitations in the previous literatures. Firstly, most studies were retrospective analysis and had a short term follow up, which gave rise to recall bias and inadequate evaluation of the effectiveness of P-ESWL. In addition, according to the studies about analysing risk factors about adverse events after P-ESWL, not all potential risk factors were enrolled in the risk factor analysis. Last but not least, more means to prevent adverse events after P-ESWL should be proposed. In reference to urinary ESWL, Tailly et al. advocate installing standard incorporation of an optically controlled coupling system in lithotripters to decrease the energy loss caused by air bubbles in the coupling interface, which can eventually prevent tissue injury [49]. In addition, an ultrasound-based, real-time stone tracking system has been used in urinary ESWL to decrease stone misidentification. When the tracking system identifies stones, it is activated and then makes the shock wave generator track and send out shock waves to the stone. When stones move out of the 2-dimensional ultrasound scan plane and cannot be identified, the tracking system would fail, and no shock wave could be sent out until stones could be identified next time [50, 51]. These two technological improvements will decrease the risks and severity of post-ESWL adverse events in urinary stones, but there are no reports for pancreatic stones. We expect a breakthrough in preventing adverse events after P-ESWL in the future.

## Data Availability

Not applicable.

## Abbreviations

CP: chronic pancreatitis
ERCP: endoscopic retrograde cholangiopancreatography
P-ESWL: pancreatic extracorporeal shockwave lithotripsy
MPD: main pancreatic duct
TAEs: transient adverse events
CT: computed tomography
DSA: digital subtraction angiography
